# Ten Simple Rules for Organizing a Scientific Meeting

**DOI:** 10.1371/journal.pcbi.1000080

**Published:** 2008-06-27

**Authors:** Manuel Corpas, Nils Gehlenborg, Sarath Chandra Janga, Philip E. Bourne

**Affiliations:** 1 European Bioinformatics Institute, Wellcome Trust Genome Campus, Hinxton, Cambridge, United Kingdom; 2 Graduate School of Life Sciences, University of Cambridge, Cambridge, United Kingdom; 3 Medical Research Council–Laboratory of Molecular Biology, University of Cambridge, Cambridge, United Kingdom; 4 Skaggs School of Pharmacy and Pharmaceutical Science, University of California San Diego, La Jolla, California, United States of America

Scientific meetings come in various flavors—from one-day focused workshops of 1–20 people to large-scale multiple-day meetings of 1,000 or more delegates, including keynotes, sessions, posters, social events, and so on. These ten rules are intended to provide insights into organizing meetings across the scale.

Scientific meetings are at the heart of a scientist's professional life since they provide an invaluable opportunity for learning, networking, and exploring new ideas. In addition, meetings should be enjoyable experiences that add exciting breaks to the usual routine in the laboratory. Being involved in organizing these meetings later in your career is a community responsibility. Being involved in the organization early in your career is a valuable learning experience [1]. First, it provides visibility and gets your name and face known in the community. Second, it is useful for developing essential skills in organization, management, team work, and financial responsibility, all of which are useful in your later career. Notwithstanding, it takes a lot of time, and agreeing to help organize a meeting should be considered in the context of your need to get your research done and so is also a lesson in time management. What follows are the experiences of graduate students in organizing scientific meetings with some editorial oversight from someone more senior (PEB) who has organized a number of major meetings over the years.

The International Society for Computational Biology (ISCB) Student Council [2] is an organization within the ISCB that caters to computational biologists early in their career. The ISCB Student Council provides activities and events to its members that facilitate their scientific development. From our experience in organizing the Student Council Symposium [3],[4], a meeting that so far has been held within the context of the ISMB [5],[6] and ECCB conferences, we have gained knowledge that is typically not part of an academic curriculum and which is embodied in the following ten rules.

## Rule 1: The Science Is the Most Important Thing

Good science, above all else, defines a good meeting; logistics are important, but secondary. Get the right people there, namely the best in the field and those who will be the best, and the rest will take care of itself. When choosing a topic for your conference, map it to the needs of your target audience. Make sure that you have a sufficiently wide range of areas, without being too general. The greater the number of topics covered, the more likely people are to come, but the less time you will have to focus on particular subject matter. Emerging areas can attract greater interest; try to include them in your program as much as possible; let your audience decide the program through the papers they submit to the general call for papers. This can be done with broad and compelling topic areas such as “Emerging Trends in …” or “New Developments in …”.

## Rule 2: Allow for Plenty of Planning Time

Planning time should range from nine months to more than a year ahead of the conference, depending on the size of your event. Allow plenty of time to select your meeting venue; to call for, review, and accept scientific submissions; to arrange for affordable/discounted hotel rooms; to book flights and other transportation options to the conference. Having outstanding keynote speakers at your event will also require you contact them months in advance—the bigger the name, the more time is required.

## Rule 3: Study All Potential Financial Issues Affecting Your Event

Sponsors are usually your primary source of funds, next to the delegates' registration fees. To increase the chances of being sponsored by industry, write them a clear proposal stating how the money will be spent and what benefits they can expect to get in return. You may also want to reserve a few time slots for industry talks or demos as a way of attracting more sponsors, but be wary that the scientific flavor of the meeting is not impacted by blatant commercialism. Make sure you first approach the sponsors that match your interest topics the closest. If they say they are not interested this year, keep their contact information, as they might be able to sponsor you in future events. Approach them early rather than later in any case. The cost of your conference will be proportional to the capacity of the venue; therefore, a good estimation of the number of attendees will provide you with a good estimate of your costs. You will need to include meals and coffee breaks together with the actual cost of renting your venue. Be aware that audiovisual costs can be additional as well as venue staff—look out for hidden costs. Aside from venue-related costs, additional expenditures might include travel fellowships, publication costs for proceedings in a journal, and awards for outstanding contributors. All these issues will determine how much you need to charge your participants to attend. Map all this out on a spreadsheet and do the math. Allow for contingencies, such as currency fluctuations and world-changing events that will impact attendance. For large meetings, consider insurance against such events. Starting with a template that others have used for previous similar conferences can be a big help.

## Rule 4: Choose the Right Date and Location

Your conference needs to be as far away as possible from established conferences and other related meetings. Alternatively, you may want to organize your event around a main conference, in the form of a satellite meeting or Special Interest Group (SIG). Teaming up with established conferences may increase the chances of attracting more people (especially if this is your first time) and also save you a great deal of administrative work. If you decide to do it on your own, you should consider how easy it is to travel to your chosen location, whether it has a strong local community in your field, and whether it has cultural or other tourist attractions. Inexpensive accommodation and airfares to your conference are always a plus.

## Rule 5: Create a Balanced Agenda

A conference is a place for people wanting to share and exchange ideas. Having many well-known speakers will raise the demand for your event (and the cost) but that has to be balanced with enough time for presentation of submitted materials. A mix of senior scientists and junior scientists always works for the better. Young researchers may be more enthusiastic and inspiring for students, while top senior scientists will be able to present a more complete perspective of the field. Allow plenty of time for socializing, too; breaks, meals, and poster sessions are ideal occasions to meet potential collaborators and to foster networking among peers.

## Rule 6: Carefully Select Your Key Helpers: the Organizing Committees

A single person will not have all the skills necessary to organize a large meeting, but the organizing committee collectively needs to have the required expertise. You might want to separate the areas of responsibilities between your aides depending on their interests and availability. Some potential responsibilities you might delegate are: 1) content and design of the Web site promoting the meeting; 2) promotion materials and marketing; 3) finance and fundraising; 4) paper submissions and review; 5) posters; 6) keynotes; 7) local organization; 8) program and speakers; 9) awards. Your organizing committee should be large enough to handle all the above but not too large, avoiding freeloaders and communication issues. It is invaluable to have a local organizing committee since they know local institutions, speakers, companies, and tourist attractions. Local organizations may also help you with administrative tasks; for example, dealing with registration of attendees and finding suitable accommodations around the venue.

## Rule 7: Have the Members of the Organizing Committees Communicate Regularly

It is good to have planning sessions by teleconference ahead of the meeting. As far as possible, everyone should be familiar with all aspects of the meeting organization. This collective wisdom will make it less likely that important issues are forgotten. The local organizers should convince everyone that the venue will work. Use these sessions to assign responsibilities ahead of the meeting. Tasks such as manning the registration tables, carrying microphones for attendees to ask questions, introducing sessions and speakers, checking presentations ahead of time, and having poster boards, materials to attach posters, etc., are easily overlooked. In short, good communication will lead to you covering all the little things so easily forgotten.

Good communication continues throughout the meeting. All organizers should be able to contact each other throughout the meeting via mobile phone and e-mail. Distribute to all organizers the names and contact information of caterers, building managers, administrative personnel, technicians, and the main conference organizer if you are having your event as part of another conference. Onsite changes that incur additional costs, however, should require the approval of a single, key organizer rather than all organizers operating independently of one another. This will ensure there are no financial surprises in the end. It is also important that you have a designated meeting point where someone from the organizing committee is going to be available at all times to help with problems.

## Rule 8: Prepare for Emergencies

Attendees need to be aware of all emergency procedures in terms of evacuation, etc. This should be discussed with the venue managers. All attendees should be reachable as far as possible during the conference. If an attendee has an emergency at home, his or her family should be able to reach them through the conference desk—mobile phones are not perfect after all.

## Rule 9: Wrap Up the Conference Properly

At the end of the conference, you should give credit to everyone who helped to make the event a success. If you have awards to present, this is the right time for the awards ceremony. Dedicate some time to thank your speakers and sponsors as well as everyone involved in the organization of the conference. Also collect feedback about the event from the delegates through questionnaires. This evaluation will help you to understand the strengths and weaknesses of your conference and give you the opportunity to improve possible future events. Have a party or some other event for all those organizing the conference.

## Rule 10: Make the Impact of Your Conference Last

Published proceedings are the best way to make the results of your conference last. Negotiate with journals far in advance of the conference to publish the proceedings. Make those proceedings as widely accessible as possible. Upload photos and videos of the event to the conference Web site and post the names of presenters who have received awards or travel fellowships. It is also a good idea to link the results of your evaluation to the Web site. Send one last e-mail to all delegates, including a summary of the activities since the conference and thanking them for their participation. This is particularly important if you are considering holding the conference again in future years, in which case include some information on your plans for the next event.

As always, we welcome your comments and experiences that you think would enrich these ten rules so that they might be useful to others. The comment feature now supported by this journal makes it easy to do this.
